# Miscellaneous Items

**Published:** 1855-05

**Authors:** 


*Miscellaneous Items.*

We copy secondarily the following items from the Nashville Journal of Medicine, in relation to a disease about which the profession is so much divided, viz.:

---

*Curability of Cancer.* By Prof. VELPEAU.

1 That no plausible reason has yet been given in favor of cancer being a disease primitively general.

2. That, on the contrary, one would be inclined to admit its title to being an affection primitively local.

3. That certain tumors of a benign nature appear to undergo, in some cases, a malignant transformation.

4. That the cause of benign and malignant tumors, adenoid and even cancerous, of the breast may probably be traced to a plastic, sanguineous, or secreted exudation into the tissues, either spontaneously or from external violence.

5. That the existence, or the non-existence, of the cancer cellule in tumors is not conclusive evidence that the disease will or will not return after operation.

6. That it would be consequently, imprudent to decide, from the evidence afforded by the microscope alone, whether to operate or not.

7. That observations and statistics are far from proving that the extirpation of tumors of the breast is always followed by a return of the disease, always useless, or even hurtful.

8. Finally, that facts sufficiently numerous, and that observations selected from my own practice, demonstrate, without the possibility of contradiction, the existence of radical cures of cancer by the operation.—*Medico-Chirurg. Review.*

---

*Applicability of Caustics to Cancer.* By Prof. VELPEAU.

The result of my experience proves that they should not be rejected absolutely as a curative means. That they are preferable to a cutting instrument. 1st. When the cancer is ulcerated in patches, and when more widely spread than deep. 2d. When, even by the cutting instrument, there is no chance of preserving a part of the integuments attacked by the tumor. 3d. When the cancer is fungating, exactly limited, and when the patient dreads much more the knife. 4th. ulcerated scirrhuss, irregular or disseminated, can be better attacked by caustics than by operation. 5th. The same may be said of ulcerated cancer adherent to the summit of axilla under the cavicle, or in the neighborhood of bone. —*Ib.*

---

*Congelation in Cancerous Returns.* By Prof. VELPEAU.

Congelation, by means of the application of pounded ice and salt, has, according to M. Velpeau, certain advantages, which may be regarded as palliative, if not, even in some cases, a means of cure. And, although the author has little experience in its application, the effects which he has seen produced would induce him to employ the frigorific mixture before entirely rejecting it, especially as a succedanum to caustics.—*Ib.*

---

*Fissures of the Anus.*—The Boston Medical Journal gives the following as a very valuable application to relieve and sometimes cure, fissures of the anus, viz.:

℞ Ext. Belladonna, iii. grs.
Ungt. Rosæ. iv. grs.

---

*Nocturnal Seminal Emissions.*—During the last two years much evidence has accumulated in the medical periodicals in favor of the efficacy of digitalis and digitaline in the treatment of involuntary seminal emissions. M. Laroche mentions a case in the Gazette Medicale of a very aggravated character which was cured by the digitaline in three weeks. The dose administered was equivalent to one grain of the pulverized leaves three times a day. We have repeatedly treated the same affections succcessfully with a mixture of equal parts of muriated tincture of iron and tincture of ergot, given in doses of thirty drops three times a day, in sweetened water.

---

*Scrofulous Intolerance of Light.*—Prof. Mauthner recommends for this troublesome affection the application to the lids of the following, viz.:

℞ Conii ½ gr.

Sweet oil, ℥i.

Mix, and apply with a hair pencil once or twice a day. We have frequently found a simple solution of morphine (4 grs. to ℥i. of water) effectual in similar cases.

---

*University of Nashville.*—Prof. J. B. Lindsley, Professor of Chemistry in the Medical Department, has recently been elected Chancellor of the University of Nashville, Tennessee.

---

*Delegates to the American Med. Association.*—Professors J. V. Z. Blaney and John Evans were appointed Delegates from Rush Medical College, and N. S. Davis from the Mercy Hospital at Chicago, to the Annual Meeting of the Association at Philadelphia on the first Tuesday in May.

---

"*Punch on Quackery.*"—Much amusement has been caused in hospital libraries by a witty but sensible article in *Punch* on the all-absorbing question of quacks and quackery. "A hand-bill has been forwarded to Mr. Punch, containing a rather choice specimen," he says, " of the lies with which the quack medicine-mongers endeavored to improve the sale of their pernicious trash. It sets forth that an Indian captain's little girl, aged eight, was in such a state of disease from dropsy, that she was given over by the doctors. Her face 'a mass of complete ulceration;' her teeth were so clenched, that her affectionate father had to wrench her mouth open with 'a wedge,' in order ' to force down a dozen pills No. 2.' The very next day she got up, dressed herself after the pills, and was 'discovered sitting on a hard form before a plate of ham and beef.' The rubbish," says Punch, " which the bill in question is intended to puff is sold (more shame to Mr. Gladstone) under a Government stamp. Government is not ashamed, for a consideration, to lend its influence to the quack. Some evening the Government, however, will be compelled to break up its partnership. In the very handbill to which we allude, and folded round the medicine, the Indian captain is made to say, 'I attribute all my ailings, weaknesses and diseases to having been bled once, and been vaccinated.' Under the stamp of the same Government," says Punch, "which introduced the Compulsory Vaccination Act, most properly enforced under Lord Palmerston's direction, is circulating a notice that all a person's ailings, weaknesses, and diseases may be attributed to vaccination! Are the people, then, to consider the Government lancet their bane—Gladstone's pills their antidote? We do think at least one thing—they have a right to complain of what Mrs. Partington would call the vaccinating policy of the Government."

---

*Physic and Music.*—Every one knows that Æsculapius was the son of Apollo, and this relationship accounts for the frequency with which we find good physicians to be good musicians also. Our annals are rich in distinguished doctors, who have had excellent voices, or who played with skill on various instruments. Boerharve was a capital performer on the flute; and it is a curious coincidence that the late great toxicologist of France, and the eminent living toxicologist of Great Britain, should have both possessed splendid bass voices. The editor of the Union Medicale comments on the number of musical confreres he meets with in the salons of Paris (Janvier 14); and we can assure him that in Edinburgh the same thing is observable. Indeed most of our public professional dinners and entertainments would now be considered dull affairs without the songs and glees with which they are enlivened by the Medical Glee Club of this city.—*Virginia Med. & Surg. Jour.*

---

*Cæsarian Section thrice upon the same Woman*, by Dr. BARJAVEL.—In a Jewess, at Carpentras, of scrofulous habit and rachitic appearance, the child presented by the feet at her first confinement. The midwife and two surgeons who attended pulled so hard, that in their efforts at delivery they separated the child's body from the head, which remained alone in the uterus. M. Barjavel (the father of the author) found, when called to the case, the cavity of the pelvis narrowed throughout by exostosis, so that no course remained but the Cæsarian section, which he performed, as usual, in the linea alba. In forty days the wound healed. In spite of the danger to which she exposed herself, she became pregnant again in three years, when the operation was again performed, and a living child was extracted. The mother suckled it, and it is now alive. In four years more she became pregnant a third time, when a young surgeon attempted the same proceeding, but he was not equally fortunate, for the child, a female, was dead, and the mother sank in a few days from hœmorrhage.—*Rev. Ther. du Midi. Dub. Med. Press.*

---

*Delirium Tremens. Tartar Emetic.*—Dr. Peddie (Monthly Journal) discountenances the treatment by opium, and recommends, from an experience of 80 cases the use of tartar emetic, in doses of from one-quarter to one-half a grain every two hours. If the bowels are not opened by this remedy, compound jalap powder is given. The patient is not to be restrained by mechanical means, and light is freely admitted into the room, as by its means optical delusions are prevented.—*Medico-Chir. Rev.*

---

*Ovarian Dropsy. Iodine.*—Dr. Simpson (Monthly Journal) refers to seven or eight cases of ovari n dropsy, in which, after tapping, tincture of iodine (two or three ounces) has been injected into the sac. In two or three cases the disease seemed arrested; but in others this was not the case. No great pain followed the injection, and no febrile symptoms, except in one case.—*Ibid.*

---

*Gonorrhœa. Subnitrate of Bismuth.*—Both in acute and chronic gonorrhœa Dr. Caby employs, three times daily, an injection, composed of water mixed with as much trisnitrate of bismuth as can be suspended. It is to be retained five minutes. It causes pain —*Ibid.*

